# Translation, cross-cultural adaptation, and validation of the HCT frailty scale for hematopoietic stem cell transplant candidates: an observational study

**DOI:** 10.1016/j.htct.2025.103933

**Published:** 2025-08-02

**Authors:** Luz Lorca, Barbara Puga Larrain, Ivana Leao Ribeiro, Ivana Gonzalez Valdivia, Angelia Fernández Hermoso, Francisca Bass Maturana, Francisco Canelo Lazcano

**Affiliations:** a Department of Physical Medicine and Rehabilitation, Hospital del Salvador, Santiago, Chile; b Department of Intensive Hematology, Hospital del Salvador, Santiago, Chile; c Faculty of Healthy Sciences, Universidad Católica del Maule, Talca, Chile; d Faculty of Healthy, Universidad Santo Tomás, Talca, Chile

**Keywords:** Hematopoietic progenitor cell transplantation, Frailty, Functionality, Validation study, HCT Frailty Scale

## Abstract

**Introduction:**

Hematopoietic stem cell transplantation (HSCT) is a treatment option for patients with hematologic malignancies. The aim of this study is to validate the Hematopoietic Cell Transplantation Frailty Scale in a Chilean population.

**Methods:**

This was a cross-sectional scale validation study. The sample consisted of patients with various hematologic malignancies who were transplantation candidates. The study had two stages: (1) translation (forward and backward) and (2) psychometric analysis, including face validity, test-retest reliability, and content validity. Descriptive analyses included mean, standard deviation, and the 95 % confidence interval. Reliability was assessed with Spearman's correlation, and content validity used Kendall's W test.

**Results:**

Fifty-four patients (53.7 % women) were included, with multiple myeloma being the most frequent diagnosis (33.3 %). Positive and strong correlations were identified (Spearman's Rho [ρ]: 1.0; *p*-value <0.001) for all items on the scale. Regarding content validity, there was agreement among evaluators for the categories of relevance and coherence (*p*-value <0.01; Kendall's W range: 0.13–0.17) but not for “clarity” (*p*-value = 0.11; Kendall's W: 0.07). Some terms in the content were adjusted without affecting the overall structure of the scale. In the retest analysis, descriptive values were similar to the initial test.

**Conclusion:**

The Spanish version of the Hematopoietic Cell Transplantation Frailty Scale for Chile is conceptually and linguistically equivalent to the original instrument. Additionally, it demonstrated adequate psychometric properties in terms of validity and reliability.

## Introduction

Hematopoietic stem cell transplantation (HSCT), is a treatment for hematological pathologies.^1^ The selection of HSCT candidates involves assessing the patient's tolerability to determine the risk of treatment-related complications, including comorbidity burden, functional status, and chronological age.^2^ Traditional pre-transplant assessment parameters, such as chronological age, comorbidity indices, and Karnofsky performance status, may fail to specifically detect the presence of frailty and functional conditions.^3^ Therefore, incorporating variables related to frailty and functionality may enhance the predictive capacity of these existing tools across all age groups, particularly in older adults.

Frailty and functionality are predictors of mortality in patients diagnosed with hematological disorders in general, and particularly in HSCT candidates.^4^ Functionality is a relevant parameter that has been correlated with survival in the older adult population in both oncological and non-oncological settings.^5^ Similarly, poor functionality has also been correlated with worse outcomes in cancer patients, particularly in HSCT recipients with poor exercise tolerance and reduced physical function.^6^^,^^7^

Likewise, frailty is common in patients undergoing HSCT and, when present, it has been associated with an increased risk of post-transplant morbidity and mortality.^8^ In this context, frailty can be present in adults of all ages and has been shown to have a negative impact on transplant outcomes,^9^ is associated with greater HSCT complexity, an increased risk of non-relapse mortality, and reduced survival.^10^

With the aim of classifying HSCT candidates, professionals at the Princess Margaret Cancer Center, Toronto, Canada, developed the Hematopoietic Cell Transplantation (HCT) Frailty Scale, a prognostic tool that is quick and easy to apply. The HCT Frailty Scale consists of eight variables, including functional assessments and laboratory tests, that allow for the categorization of HSCT candidates into three groups: “fit,” “pre-frail,” and “frail,” regardless of age.^8^^,^^10^

Currently, there are no validated scales to assess frailty and functionality in HSCT candidates in the Chilean population. Therefore, the objective of this study is to validate the HCT Frailty Scale for this population.

## Methods

### Design

An observational study with a cross-sectional design, translation, and adaptation, aimed at validating a measurement instrument that follows the guidelines of the COSMIN (COnsensus-based Standards for the selection of health Measurement INstruments) framework.^11^ This study was approved by the Scientific Ethics Committee of the Metropolitan Eastern Health Service (December 5, 2023).

### Participants

Fifty-four HSCT candidates aged ≥17 years with a diagnosis of onco-hematological diseases participated in this psychometric study in a public hospital in Santiago, Chile. Individuals with observed functional or cognitive deficits, or significant disabilities that prevented them from understanding the study, performing simple functional tests, or giving their written informed consent, were excluded. Additionally, individuals with insufficient understanding of Spanish, which hindered comprehension of instructions and evaluator directions, were also excluded.

### Procedures

Patients attending their first consultation with the hematologist in the HSCT program were recruited from the HSCT unit. Those who met the eligibility criteria were invited to participate in the study, with a detailed explanation of the objectives and procedures involved. Those who voluntarily agreed to participate signed an informed consent form prior to enrollment.

The evaluations were conducted between December 2023 and June 2024 by two physical therapists at a physical medicine and rehabilitation clinic.

The original authors authorized the use of the scale, and the study was conducted in two stages:

## Forward and backward translation

This process was carried out in the following order:

### Forward translation

The scale was first translated into Spanish by two native Chilean speakers who are bilingual in English. They worked independently during the translation process.

### Comparison and merging

The resulting translations were compared and merged into a single version by a test coordinator. Any discrepancies between the versions were analyzed and resolved by the translators and the coordinator.

### Backward translation

The scale was then translated back into English by a native English speaker (language teacher) who is bilingual in Spanish and did not participate in the translation stage.

### Comparison and evaluation

The back-translated version was compared and evaluated in terms of similarities and conceptual equivalence with the version obtained in phase 1.2 and, in parallel, with the original scale.

### Final consensus meeting

**Figure 1:**
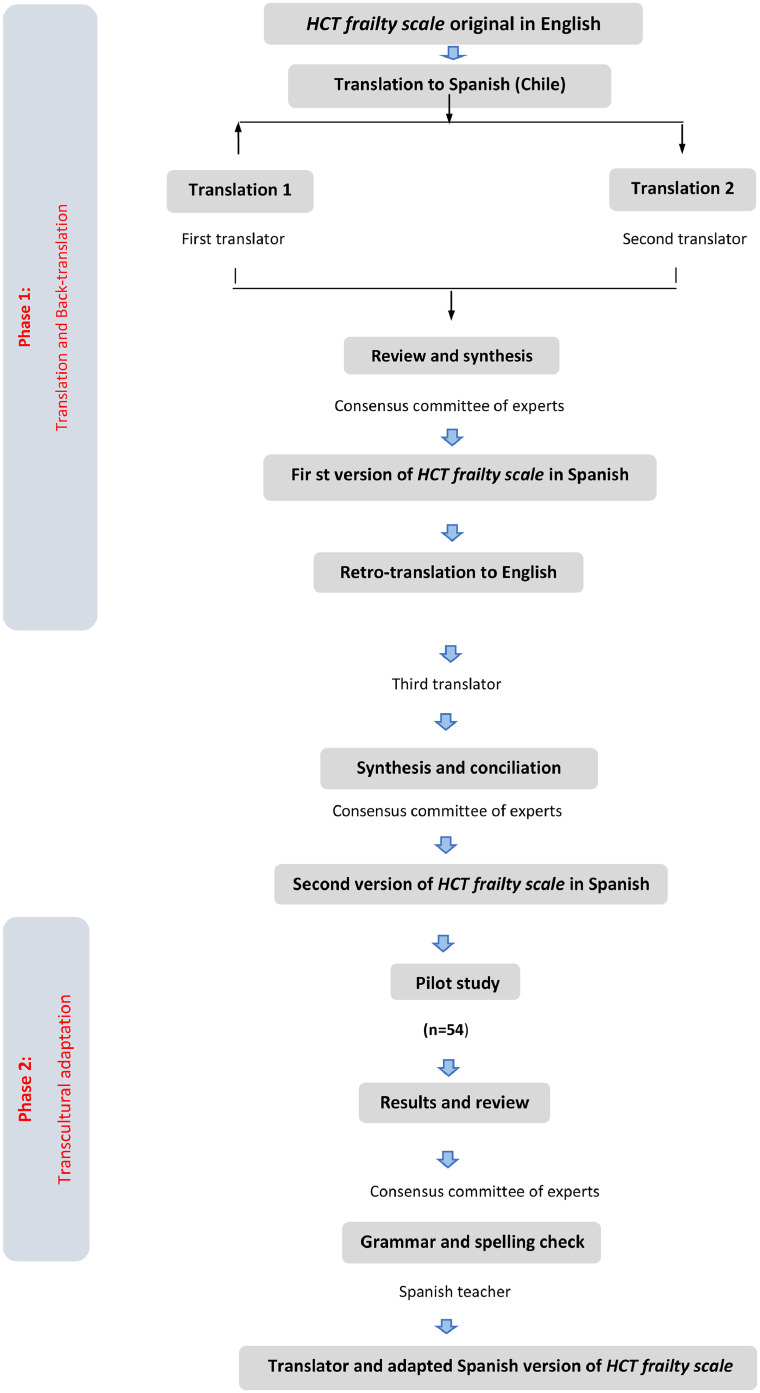
Flowchart of the translation and cross-cultural adaptation phases.

In a consensus meeting of the researchers and translators, a second unified version was obtained that was consistent with the original version, with minor adjustments made for the Spanish scale tailored for Chile. Finally, through consensus, a final version derived from the previous process was sent to the original authors for review. After some corrections, they approved the final version to be applied in a second pilot testing phase (Figure 1).

## Psychometric properties analysis

### Apparent validity

Since the version obtained in the first stage could not be limited to a simple translation, conceptual and semantic equivalence must be ensured between the original version and the adapted version, as well as the understanding of the obtained version by the target population. In this stage, the degree to which the content of the scale adequately reflects the construct to be measured was assessed. For this purpose, a pilot test was conducted with 54 patients, with some guiding questions being applied. The scale was first administered to 27 patients, observations were compiled, and necessary changes were made. Subsequently, the remaining 27 patients were evaluated, and new observations were gathered.

### Reproducibility (test-retest reliability)

In this stage, the stability of the scale over time was evaluated by administering it at two different timepoints. The scale was applied twice by two physical therapists to a group of 30 patients, with a 24-h interval between assessments. To improve data reliability and facilitate interpretation, the two assessments were conducted within a maximum interval of 24 h, as recommended by the reviewers.

This interval was chosen to ensure a sufficient period of time to minimize the risk of progressive physical changes in the patients.

### Content validity (face validity)

Content validity assessed whether the scale made sense to the professionals who care for HSCT candidates. Twenty-one professionals from different HSCT care centers nationwide (hematologists, physiotherapists, and nurses) with at least 5 years of experience in hematology and HSCT patient care were consulted. For content validity, an individual method was used, involving a written survey that each participant answered without having contact with the others. The scale was evaluated in terms of “coherence,” “clarity,” and “relevance” for each of the eight items composing the scale. A Likert-type survey with five response alternatives was used: “Strongly agree,” “Agree,” “Neither agree nor disagree,” “Disagree,” and “Strongly disagree” for each statement. An observation section was also included for additional information.

## Instruments used

### Hematopoietic Cell Transplantation *Frailty Scale*

This scale was developed by professionals at the Princess Margaret Cancer Centre and is designed to classify patients who are HSCT candidates. It consists of eight items, which include various subjective and objective tests and scales. These items were carefully selected and appropriately modified based on previous studies conducted in older populations and transplant centers.^8^^,^^10^

The items are: Clinical Frailty Scale (CFS) score^12^; Instrumental Activities of Daily Living (IADL) ^13^; Self-Rated Health Questionnaire (SRH-Q)^14^; Fall Risk Assessment (Falls-test); Grip Strength (Dynamometry)^15^; Timed Up and Go test (TUGT)^16^; and Laboratory Tests such as serum albumin^17^ and C-reactive protein (CRP).^18^ Each variable is scored as either “normal” (0 points) or “abnormal” (1, 1.5, or 2 points, depending on the specific variable and its defined cut-off value). The total score is derived from the total of individual item scores, yielding a possible range of from 0 to 10.5 points. This allows for the classification of HSCT candidates in three categories: “fit,” “pre-frail,” and “frail,” regardless of age and underlying diagnosis.

In the present study, a hydraulic dynamometer was used for the grip strength test (Jamar®, J A Preston Corporation, New York, USA).

### Performance status

This variable was assessed using the Karnofsky Performance Status (KPS) scale, a numerical scale from 0 to 100. A lower score indicates a worse performance status.^19^ In this study, the ranges used were: 50–60, 70–80, and 90–100.

### Sociodemographic and clinical background

Data was collected on sociodemographic and clinical factors such as age, sex, education level, marital status, employment status, smoking and alcohol drinking habits, weight, height, diagnosis, type of HSCT, treatments received, Disease Risk Index (DRI), and the Hematopoietic Cell Transplantation-Comorbidity Index (HCT-CI).^20^

### Statistical analysis

The data were tabulated and analyzed using the Statistical Package for Social Sciences (SPSS) version 25.0. Descriptive analyses were conducted, considering the mean, standard deviation, and the 95 % confidence interval. Spearman’s correlation test was used for reliability analysis between the two assessments considering the items of the scale in the test-retest. The following values were considered for interpretation: between 0.00 and 0.10, insignificant correlation; between 0.10 and 0.39, weak correlation; between 0.40 and 0.60, moderate correlation; between 0.70 and 0.89, strong correlation; and between 0.90 and 1.00, very strong correlation.^21^

Content validity was determined using Kendall's W test, considering the dimensions of “clarity,”, “coherence,” and “relevance” for each item of the scale based on data from expert evaluators. The following interpretation was applied: 0: No agreement; 0.10: Weak agreement; 0.30: Moderate agreement; 0.60: Strong agreement; and 1.0: Perfect agreement.^22^

## Results

### Translation and cross-cultural adaptation

After the forward translation process carried out by two independent translators, the versions were compared and deliberations took place to determine which words should be adjusted for better understanding, resulting in a single version (Table 1), which then proceeded to the back-translation process. Subsequently, the back-translated version was compared with the original version, and no significant differences were found, confirming that the translations were similar.

**Table 1:** Forward translation results.

Item	Original version HCT-Frailty Scale	First Spanish translation	Second Spanish translation	Final agreed version
1	Clinical frailty score (CFS): ≥ 3(frail) [vs 1–2 (no frail)]	Puntaje de fragilidad clínico (PCF): ≥ 3 (frágil) [vs 1–2 (no frágil)]	Puntuación clínica de fragilidad (PCF): ≥ 3 (frágil) [vs 1–2 (no frágil)]	Puntaje clínico de fragilidad (PCF): ≥ 3 (frágil) [vs 1–2 (no frágil)]
2	Instrumental Activities of daily living (IADL) score: ≥1 limitation [vs no limitation]	Actividades instrumentales de la vida diaria (AIVD)puntaje: ≥ 1 Limitación [vs sin limitación]	Puntaje Actividades instrumentales de la vida diaria (AIVD): ≥ 1 Limitación [vs sin limitación]	Puntaje en Actividades instrumentales de la vida diaria (AIVD): ≥ 1 Limitación [vs sin limitación]
3	Time and go test (TUGT): Abnormal> 10 seg. [vs normal]	Prueba de levantarse y caminar cronometrada: Anormal > 10 seg. [vs normal]	Test de tiempo de levantarse y caminar: Anormal > 10 seg. [vs normal]	Prueba de levantarse y caminar cronometrada: Anormal> 10 seg. [vs normal]
4	Grip Strength (GS): Abnormal [vs normal] If female <16 kg. If male <26 kg.	Fuerza de agarre (FA): Anormal [vs normal] Si es mujer menos de 16 kg. Si es hombre menos de 26 kg.	Fuerza de prensión manual (FPM): Anormal [vs normal] Si es mujer menos de 16 kg. Si es hombre menos de 26 kg.	Fuerza de prensión manual (FPM:) Anormal [vs normal]. Si es mujer menos de 16 kg. Si es hombre menos de 26 kg.
5	Self-rated Health question(SRH-Q): Fair, poor (vs excellent, very good, good)	Pregunta sobre autopercepción de salud (PAS): Regular,mala (vs excelente,muy buena,buena)	Pregunta auto informada de salud (PAS): Regular,mala (vs excelente,muy buena,buena)	Pregunta auto informada de salud (PAS): *Se le pide al paciente que califique su salud actual en comparación con otras personas de su edad entre*: Regular, mala (vs excelente,muy buena,buena)
6	Falls in last 6 months Yes (vs no)	Caídas en los últimos 6 meses Sí (v no)	Caídas los últimos 6 meses Sí (vs no)	Caídas los últimos 6 meses Sí (vs No)
7	Albumin serum level (Alb): Abnormal (<38 g/L) [vs normal]	Nivel de albumina sérica (Alb): Anormal (<38 g/L) [vs normal]	Nivel de albumina sérica (Alb): Anormal (<38 g/L) [vs normal]	Nivel de albumina sérica (Alb): Anormal (<38 g/L) [vs normal]
8	C-reactive protein (CRP): Abnormal(≥11 mg/L) [vs normal]	Proteína C reactiva (PCR): Anormal (≥11 mg/L) [vs normal]	Proteína C reactiva (PCR): Anormal (≥11 mg/L) [vs normal]	Proteína C reactiva (PCR): Anormal (≥11 mg/L) [vs normal]
	*Total score*	***Puntuación total***	***Puntaje total***	***Puntaje total***
	*Patient risk classifications*	***Clasificación de riesgo del paciente***	***Categorización de riesgo del paciente***	***Categorización del paciente***

In the section of instructions for applying the scale, there were differences in the translation of the word “test,” where the first translator translated it as “prueba,” and the second translator kept it as “test,” with the final consensus being “prueba.” Similarly, in the application instructions, the word “fit” in the original version was translated into Spanish as “apto.” In the back-translation, it was rendered as “suitable,” but it was consensually accepted as “fit.”

### Psychometric properties

A total of 54 HSCT candidates participated in the psychometric evaluation of the scale. The median age was 36.9 ± 14.6 years, with the majority being women (53.7 %) and multiple myeloma being the most prevalent diagnosis (33.3 %). Twelve patients (12.2 %) were categorized as “fit,” 26 (48.1 %) as “pre-frail,” and 16 (29.6 %) as “frail.” The sociodemographic and clinical background of the participants are shown in Table 2.

**Table 2:** Participant characterization for face validity (*n* = 54).

Variable	
Sex - *n* (%) Female Male	29 (53.7) 25 (46.3)
Age – years	36.9 ± 14.6 (32.9–40.9)^a^
Height - m	1.64 ± 0.10 (1.62–1.67) a
Weight – kg	73.8 ± 16.7 (69.2–78.4) a
Body Mass Index - kg/m²	27.1 ± 5.0 (25.7–28.4) a
Educational level - *n* (%)	
Primary Secondary Technical University	7 (13.0) 22 (40.7) 14 (25.9) 11 (20.4)
Marital status - *n* (%)	
Single Married Cohabiting Widowed Divorced	32 (59.3) 16 (29.6) 2 (3.7) 1 (1.9) 3 (5.6)
Employment status - *n* (%)	
Employed On medical leave	6 (11.1) 22 (40.7)
Unemployed	7 (13.0)
Other (student or homemaker)	19 (35.2)
Drinking habit - *n* (%) No Occasionally	17 (31.5) 37 (68.5)
Smoking habit - *n* (%) No Yes Former smoker	27 (50.0) 8 (14.8) 19 (35.2)
Diagnosis - *n* (%)	
Multiple myeloma Hodgkin lymphoma Non-Hodgkin lymphoma Acute lymphoblastic leukemia Acute myeloid leukemia Myelodysplastic aplasia Hypoplastic myelodysplastic syndrome	18 (33.3) 9 (16.7) 5 (9.3) 13 (24.1) 4 (7.4) 4 (7.4) 1 (1.9)
Type of treatment - *n* (%)	
Chemotherapy/Radiotherapy/Immunotherapy Chemotherapy/Immunotherapy Chemotherapy/Radiotherapy Chemotherapy Immunotherapy Not declared	1 (1.9) 5 (9.3) 5 (9.3) 39 (72.2) 3 (5.5) 1 (1.8)
Type of HPCT - *n* (%)	
Autologous Allogeneic-MRD (matched related donor) Allogeneic-Haploidentical	31 (57.4) 5 (9.3) 18 (33.3)
DRI - *n* (%) Low Intermediate High Very high Not evaluable	6 (11.0) 35 (64.8) 11 (20.4) 1 (1.9) 1 (1.9)
Karnofsky - *n* (%) 50–60 70–80 90–100	6 (11.1) 34 (73.0) 14 (25.9)
HSCT-CI - *n* (%) 0 1–2 ≥ 3 Not evaluable	32 (59.3) 14 (25.9) 5 (9.3) 3 (5.6)
Categorization of patients according to the HCT Frailty Scale - *n* (%) Frail Pre-frail Fit	12 (22.2) 26 (48.1) 16 (29.6)

MRD: matched related donor; DRI: disease risk index. HPCT: Hematopoietic Progenitor Cell Transplantation; HSCT-CI: Hematopoietic Cell Transplantation-Specific Comorbidity Index.

a Mean ± standard deviation (95 % confidence interval).

### Apparent validity

The first 27 patients evaluated stated that the scale was easy to understand, except for Item 6, “self-reported health question” which required further explanation for 12 patients. Regarding the functional tests, they mentioned that they were not difficult to perform. They also reported that the instructions were clear and the items were relevant and appropriate for assessing their frailty and functionality before the HSCT. No modifications were made during this stage.

Later, in the second round, the scale was applied to another 27 patients, of whom two also had some difficulty answering Item 6. Four patients experienced some difficulty executing the TUGT. All 54 evaluated patients emphasized the importance of being assessed on their “functional status” as a critical aspect prior to the transplant.

### Reliability (test-retest)

Regarding the reliability analysis between the two assessors (test-retest), positive and strong correlations were identified (Spearman's Rho [ρ]: 1; *p*-value <0.001) for all items of the scale (Table 3).

**Table 3:** Test-retest reliability analysis of the Hematopoietic Cell Transplantation Frailty Scale (*n* = 30).

Dimension	Item	Spearman's Rho
Clinical Frailty Scale	1	1.00^a^
Instrumental activities of daily living (IADL)	2	0.93^a^
Timed and go test (TUGT)	3	0.97^a^
Handgrip strength	4	0.92^a^
Self-reported health question	5	1.00^a^
Falls in the last 6 months	6	1.00^a^
Albumin level	7	1.00^a^
C-reactive protein (CRP)	8	1.00^a^

a *p*-value <0.01.

### Content validity

In general, all the variables of the scale were evaluated as consistent and relevant (Kendall's W range: 0.13–0.17; *p*-value <0.05). However, there were discrepancies regarding the clarity of some items (Kendall's W: 0.07; *p*-value = 0.11; Table 4).

**Table 4:** Content validity and inter-rater agreement on the “clarity,” “consistency,” and “relevance” of the items in the Hematopoietic Cell Transplantation Frailty Scale (*n* = 21).

		Mean	Standard deviation	Minimum	Maximum	W Kendall	
	Item					Range	Kendall’s W (*p*-value)
Clarity	1	4.9	0.21	4.0	5.0	4.5	0.07 (0.11)
	2	4.9	0.21	4.0	5.0	4.5	
	3	4.9	0.21	4.0	5.0	4.5	
	4	4.9	0.21	4.0	5.0	4.5	
	5	4.9	0.30	4.0	5.0	4.3	
	6	4.8	0.35	4.0	5.0	4.1	
	7	5.0	0.00	5.0	5.0	4.7	
	8	5.0	0.00	5.0	5.0	4.7	
Consistency	1	4.9	0.21	4.0	5.0	4.6	0.13 (<0.01)
	2	4.9	0.30	4.0	5.0	4.4	
	3	4.9	0.21	4.0	5.0	4.6	
	4	4.9	0.21	4.0	5.0	4.6	
	5	4.9	0.30	4.0	5.0	4.4	
	6	4.7	0.43	4.0	5.0	3.8	
	7	5.0	0.00	5.0	5.0	4.7	
	8	5.0	0.00	5.0	5.0	4.7	
Relevance	1	4.9	0.21	4.0	5.0	4.5	0.17 (<0.01)
	2	4.9	0.21	4.0	5.0	4.5	
	3	4.9	0.21	4.0	5.0	4.5	
	4	4.9	0.21	4.0	5.0	4.5	
	5	4.9	0.21	4.0	5.0	4.5	
	6	4.7	0.43	4.0	5.0	3.8	
	7	5.0	0.00	5.0	5.0	4.7	
	8	5.0	0.00	5.0	5.0	4.7	

Based on the analysis and observations made by the experts, improvements were incorporated to enhance the clarity and understanding of the scale, and some changes were made to the version from the first stage.

For Item 3, it was agreed to use the TUGT without translation, as this test is widely recognized and accepted, and has been integrated by professionals in the national clinical context.

For Item 4, which evaluates handgrip strength, some experts noted that while the test is coherent and relevant for this population, they inquired about the appropriateness of using the scale with values adjusted for the Chilean population. The original authors argued that the cutoff points (16 kg for women and 26 kg for men) used in the scale’s design methodology, supported by previous studies, were specifically chosen to make the scale applicable to other institutions. Therefore, the original values were retained.

Additionally, to improve comprehension, the phrase “patient classification” was changed to “patient categorization.” Furthermore, for the CFS scoring, the original version mentioned that it should be performed by “a physician,” which was changed to “healthcare professional” to adjust to the national clinical context, providing the option for these assessments to be conducted by other professionals.

A final version of the HCT-Frailty Scale adapted for use in Chile is provided in Supplementary Material 1.

There were also differences regarding the time required for application. The original authors mentioned 5–6 min, which was insufficient, as professionals took between 20–25 min to complete the scale. Additionally, it was consensually deliberated that the most suitable professionals for administering the scale are physiotherapists, as they frequently conduct all the tests that make up the scale in various clinical settings.

**Figure 2:**
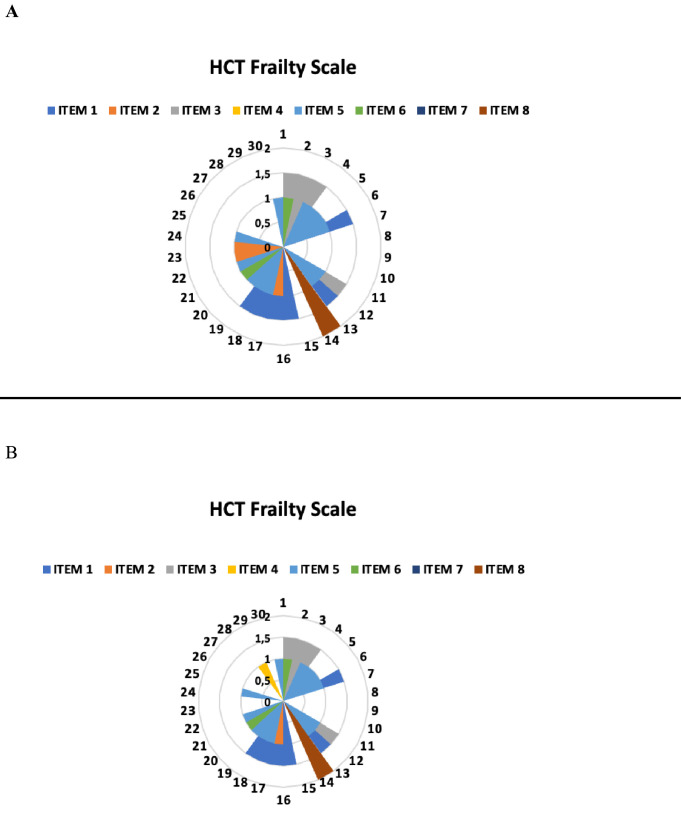
Results of the test with the Hematopoietic Cell Transplantation Frailty Scale - A: Test; B: Retest.

Regarding the results of each item on the Frailty-Functionality Scale reported by participants in the test and retest evaluations (Figure 2).

## Discussion

This study resulted in a Spanish (Chile) version of the HCT Frailty Scale, which was culturally adapted for the Chilean population after a process of translation, back-translation, and evaluation of apparent validity in patients undergoing HSCT. The translation and cultural adaptation process aimed to produce a version of the HCT Frailty Scale that maintains equivalent semantic, conceptual, and technical levels as the original instrument, ensuring that it can be understood by individuals when evaluating their functional status and frailty in their local context.^23^

To our knowledge, this is the first validated version for Spanish-speaking individuals in Latin America and could serve as a reference for its use in these countries. However, it is recommended that before using this version of the scale, the authors conduct a thorough review for cultural adaptation and linguistic validation.^24^^,^^25^ Although the main mission of the Royal Spanish Academy (Real Academia Española) is to ensure that changes in the Spanish language do not break its essential unity, there are certain nuances and terminology preferences in each Spanish-speaking country.

Regarding the apparent validity of the HCT Frailty Scale, it was found to be appropriate for assessing the construct in HSCT candidates. Patients reported that the version was clear and easy to understand. They also highlighted the relevance of being evaluated on their “frailty and functionality” condition as a critical aspect prior to transplantation.

Similarly, for clinical use, scales require valid, reproducible, and reliable evaluation methods. In this study, the reliability analysis through test-retest showed that the Spanish (Chile) version of the HCT Frailty Scale has adequate reliability in terms of information stability.

Regarding content validity according to the consulted experts, the results of this study indicate that the eight items of the scale are relevant and consistent for evaluating the construct “frailty and functionality” in HSCT candidates, but not for the dimension of clarity. Considering these results and the qualitative input provided by the experts in the observations section, some changes were made to the Spanish version of the scale to improve aspects related to clarity. These changes were made because the benefits derived from these suggestions aim to enhance the validity of the scale, as they directly impact the content of the items and certain aspects related to its structure, thereby avoiding potential content biases and/or errors during subsequent application, as mentioned by some authors.^26^^,^^27^

In general, the professionals reported that the scale was easy to apply and they were confident that they had understood the instructions correctly. However, they noted that more time was needed than the 5–10 min stipulated by the original authors of the scale, as it involves several items that require precision, along with functional tests that necessitate additional “learning” time for patients who are performing the tests for the first time. Additionally, a significant number of these patients experience substantial functional deterioration prior to HSCT, a condition that may limit their performance in functional tests.^2^^,^^28^^,^^29^

Moreover, it is suggested that for better understanding and to facilitate the application of the scale, training and the development of a support manual for healthcare professionals who will assess these patients should be provided.

One limitation of this study was the lack of published psychometric studies for other countries using the HCT Frailty Scale, which prevented the possibility of making broader comparisons with these results.

Furthermore, this study had a small sample size, which is inherent to the type and objective of the study. However, as this study represents an initial step in the evaluation and application of the scale, ongoing research is focused on analyzing other psychometric properties of the Spanish version of the HCT Frailty Scale in a larger patient sample.

The use of the validated HCT Frailty Scale is important for assessing the true extent of frailty and functionality in this population, which could enable the proposal of pre-transplant interventions, such as pre-habilitation, for patients who are not “fit.”^1^^,^^29^

A key strength of the study is that it proposes a scale the application of which does not require additional costs and can be implemented using existing resources. Additionally, this study recruited a nationally representative sample, as patients from across the country participated, considering that the Hospital del Salvador is a national referral center for HSCT.

## Conclusions

The Spanish version of the HCT Frailty Scale for Chile is conceptually and linguistically equivalent to the original instrument. Furthermore, it demonstrated adequate psychometric properties in terms of validity and reliability. Therefore, it is recommended for clinical use to categorize patients who are HSCT candidates.

## Funding

No funding was received to assist with the preparation of this manuscript.

## Ethical approval

Approved by the Scientific ethics committee of the Metropolitan East Health Service on December 5, 2023.

## Data availability

Data supporting the results can be accessed by previous requires to the corresponding author.

## Conflicts of interest

The authors declare they have no financial interests.

## Appendix Supplementary materials

Image, application 1
