# Supplementary material for: Translation, cross-cultural adaptation, and validation of the HCT frailty scale for hematopoietic stem cell transplant candidates: an observational study
Source: Hematol Transfus Cell Ther. 2025 Aug 2;47(3):103933. doi: 10.1016/j.htct.2025.103933 (PMC12337121; doi:10.1016/j.htct.2025.103933)
Supplement: Supplementary file 1 [file mmc1.docx]

| **ITEM** | **PUNTAJE** |
| --- | --- |
| Puntaje clínico de fragilidad (PCF) |  |
| Puntaje actividades instrumentales de la vida diaria (AIVD) |  |
| Time and go test (TUGT) |  |
| Fuerza de prensión manual |  |
| Pegunta de salud auto informada |  |
| Caídas los últimos 6 meses |  |
| Nivel de albúmina sérica |  |
| Proteína C reactiva |  |
| **Puntaje Total**  (0-10.5) |  |
| **Apto**  (≤ 1)  **Categorización Pre-frágil**  (>1< 5.5)  **Frágil**  (≥ 5.5) |  |

| **Ítem** | **Descripción** | **Puntaje** |
| --- | --- | --- |
| Puntaje clínico de fragilidad (PCF) | ≥ 3 (frágil)  1-2 (no frágil) | 1.5 puntos  0 puntos |
| Puntaje en Actividades instrumentales de la vida diaria (AIVD) | ≥ 1 Limitación  Sin limitación | 1 punto  0 puntos |
| Time and go test (TUGT)  *(Prueba de levantarse y caminar cronometrada)* | Anormal> 10 seg.  Normal: ≤ 10 seg. | 1.5 puntos  0 puntos |
| Fuerza de prensión manual (FPM) | Anormal:  Si es mujer, menos de 16 kg.  Si es hombre, menos de 26 kg.  Normal | 1 punto  1 punto  0 puntos |
| Pregunta auto informada de salud (PAS) | *Se le pide al paciente que califique su salud actual en comparación con otras personas de su edad entre:*  Regular, mala  excelente, muy buena, buena | 1 punto  0 puntos |
| Caídas los últimos 6 meses | Sí  No | 1 punto  0 puntos |
| Nivel de albumina sérica (Alb) | Anormal (<38g/L)  Normal | 1.5 puntos  0 puntos |
| Proteína C reactiva (PCR) | Anormal (≥11 mg/L)  Normal | 2.0 puntos  0 puntos |
|  | ***Puntaje total:***  Se obtiene de la sumatoria de los puntos de cada ítem. |  |

**HCT frailty scale**
